# Akhirin Functions as an Innate Immune Barrier to Preserve Neurogenic Niche Homeostasis During Mouse Brain Development

**DOI:** 10.3390/cells15020151

**Published:** 2026-01-14

**Authors:** Mikiko Kudo, Tenta Ohkubo, Taichi Sugawara, Takashi Irie, Jun Hatakeyama, Shigehiko Tamura, Kenji Shimamura, Tomohiko Wakayama, Naoki Matsuo, Kinichi Nakashima, Takahiro Masuda, Kunimasa Ohta

**Affiliations:** 1 Division of Molecular Neuroimmunology, Medical Institute of Bioregulation, Kyushu University, Fukuoka 812-8582, Japan; takahiro.masuda@bioreg.kyushu-u.ac.jp; 2 Department of Stem Cell Biology, Graduate School of System Life Sciences, Kyushu University, Fukuoka 819-0395, Japan; tenta-o@outlook.jp; 3 Department of Histology, Graduate School of Medical Sciences, Kumamoto University, Kumamoto 860-8556, Japan; tsugawara@kumamoto-u.ac.jp (T.S.); twaka@kumamoto-u.ac.jp (T.W.); 4 Department of Stem Cell Biology and Medicine, Graduate School of Medical Sciences, Kyushu University, Fukuoka 812-8582, Japan; irie.takashi.258@m.kyushu-u.ac.jp (T.I.); nakashima.kinichi.718@m.kyushu-u.ac.jp (K.N.); 5 Department of Neurology, Neurological Institute, Graduate School of Medical Sciences, Kyushu University, Fukuoka 812-8582, Japan; 6 Department of Brain Morphogenesis, Institute of Molecular Embryology and Genetics, Kumamoto University, Kumamoto 860-8556, Japan; jhatakey@kumamoto-u.ac.jp (J.H.); simamura@kumamoto-u.ac.jp (K.S.); 7 Department of Molecular Cell Biology, Faculty of Arts and Science, Kyushu University, Fukuoka 819-0395, Japan; stamura@artsci.kyushu-u.ac.jp; 8 Department of Biology, Faculty of Sciences, Kyushu University, Fukuoka 819-0395, Japan; matsuo.naoki.722@m.kyushu-u.ac.jp; 9 Department of Stem Cell Biology, Faculty of Arts and Science, Kyushu University, Fukuoka 819-0395, Japan

**Keywords:** Akhirin, NSC/NPC niche, embryonic neurogenesis, neurogenic niche homeostasis, brain innate immune barrier, brain development

## Abstract

**Highlights:**

- Akhirin (AKH) is secreted by choroid plexus epithelial cells and localizes to the ventricular surface during embryonic brain development.
- Loss of AKH disrupts neural stem cell homeostasis by inducing inflammatory cytokines in the cerebrospinal fluid.
- AKH is cleaved under inflammatory conditions, and its LCCL domain directly binds bacteria, providing innate immune protection in the developing neurogenic niche.
- AKH functions as a barrier molecule that integrates immune defense with the maintenance of neural stem cell niche integrity during early neurodevelopment.

**Abstract:**

Neurogenesis is tightly regulated by complex interactions among neural stem and progenitor cells (NSCs/NPCs), blood vessels, microglia, and extracellular matrix components within the neurogenic niche. In the embryonic brain, NSCs reside along the ventricular surface, where cerebrospinal fluid (CSF) directly regulates their proliferation. Here, we identify Akhirin (AKH) as a critical regulator that preserves the integrity of the NSC niche during mouse brain development. At embryonic day 14.5, AKH is secreted and enriched at the apical surface of choroid plexus epithelial cells and the ventricular lining. Loss of AKH leads to increases the inflammatory cytokine expression in the CSF and disrupts NSC niche homeostasis. Furthermore, AKH is cleaved upon inflammatory stimulation, and its LCCL domain directly binds bacteria, thereby preventing their spread. These findings reveal that AKH functions as a protective barrier molecule within the developing neurogenic niche, providing immune protection and preserving NSC niche homeostasis during periods when the innate immune defenses are still immature.

## 1. Introduction

A fundamental event in brain development is neurogenesis, the generation of new neurons from neural stem cells and neural progenitor cells (NSCs/NPCs). The region surrounding NSCs/NPCs, known as the neurogenic niche, plays a central role in regulating neurogenesis during both embryonic and adult stages [1,2,3]. The subventricular zone (SVZ), located adjacent to the lateral ventricle (LV), is one of the major neurogenic niches in which neurogenesis persists throughout life in mice [4,5,6]. During embryonic development, the periventricular layer surrounding the ventricles serves as a key NSC/NPC niche that supports the production of neurons destined for the cerebral cortex.

The NSC/NPC niche comprises not only NSCs/NPCs and neurons but also blood vessels, microglia, and extracellular matrix (ECM) components secreted by these cells. During early brain development, the NSC/NPC niche directly interfaces with the ventricular lumen, rendering it highly responsive to bioactive molecules secreted into the cerebrospinal fluid (CSF). These CSF-derived signals critically regulate the proliferation, differentiation, and survival of NPCs [7,8]. At early developmental stages, the ependymal cell layer, which normally separates the brain parenchyma from the ventricular space, has not yet fully differentiated, leaving NSCs directly exposed to the ventricular lumen [9]. While the role of ECM components in niche regulation is well documented [10], the specific contributions of CSF-derived factors on NSC regulation remain poorly understood [8].

The CSF is produced by choroid plexus (ChP), which is composed of capillaries and ChP epithelial cells. Tight junctions between ChP epithelial cells form the blood–CSF barrier, which selectively controls the passage of substances from the blood into the CSF. The ChP forms shortly after neural tube closure, and CSF production begins prior to the complete development of the brain vasculature. In addition to producing CSF, the ChP supplies nutrients to the developing brain through the transport of various substances from the blood to the CSF [11,12].

Previously, we identified Akhirin (AKH) as a novel secreted protein expressed in the ciliary marginal zone and lens epithelium of the developing chick eye [13,14]. AKH contains one Limulus factor C, Coch-5b2, and Lgl1 (LCCL) domain and two von Willebrand factor (vWF) domains, shows high sequence homology to mouse Cochlin, and exhibits heterophilic cell adhesion activity [15]. We later reported that AKH is robustly expressed in the NSC niche regions of the spinal cord and brain at birth, whereas its expression declines and becomes undetectable by postnatal day 30 (P30) [14,15,16]. We also demonstrated that AKH regulates NSCs proliferation and differentiation during spinal cord and brain development [15,16,17]. However, how AKH contributes to the regulation of NSCs/NPCs proliferation and brain formation remains unclear.

Here, we report that AKH regulates CSF composition and NSC/NPC niche homeostasis by functioning as a barrier molecule within the ChP. We further show that AKH in the fetal ventricular system is cleaved following activation of the maternal immune system and acts to restrict the spread of infection. Together, these findings suggest that AKH plays a previously unrecognized and critical role in barrier formation during embryonic development, providing immune protection and supporting normal brain development when innate immune defenses are still immature.

## 2. Materials and Methods

### 2.1. Animals

The generation of AKH knockout mice (AKH^−^/^−^) has been described previously [15,16]. All mice were maintained on a mixed C57BL/6 genetic background, and both male and female mice were used. Animals were housed under a 12-hour(h) light/12 h dark cycle and ad libitum access to food and water. All efforts were made to minimize animal suffering and to reduce the number of animals used. No animals were excluded or dropped out from study. All mice subjected to the experimental procedures were included in the final analyses, as every effort was made to ensure successful surgery and postoperative care, thereby minimizing animal loss. All experiments on mice were performed in accordance with the guidelines of the Committee on Animal Research at Kyushu University.

### 2.2. Brain Tissue Processing for Histology

Mice were anesthetized deeply and perfused transcardially with phosphate-buffered saline (PBS) and 4% paraformaldehyde (PFA) in PBS to fix the brain tissue. Samples were fixed in 4% PFA at 4 °C overnight. For cryosectioning, samples were 30% sucrose protected at 4 °C for 2 days and transferred Optimal Cutting Temperature (OCT) (Tissue-Tek^®^; Sakura Finetek, Torrance, CA, USA) at room temperature (RT) for 1 h. Samples and frozen immediately at −80 °C. Coronal sections were cut in 30 μm of thickness by cryostat (Leica Microsystems, Wetzlar, Germany).

### 2.3. Mouse Model of Ischemic Stroke

Transient middle cerebral artery occlusion (tMCAO) was performed as described previously [18]. Briefly, 8-week-old mice were anesthetized with a mixture of 4 mg/kg midazolam, 0.3 mg/kg medetomidine, and 5 mg/kg butorphanol, and tMCAO was generated by the insertion of a silicon-coated monofilament (6–0 medium MCAO suture L34 No. 602334, Doccol Corporation, Sharon, MA, USA) via the right proximal external carotid artery into the internal carotid artery. The distance from the suture tip to the right common carotid artery bifurcation was 9–10 mm. Thirty minutes after the right tMCAO, the inserted monofilament was withdrawn to allow reperfusion. After the operation, the effects of medetomidine were reversed with atipamezole. To prevent intraoperative hypothermia, body temperature was maintained from surgery until recovery from anesthesia using a heating mat and thermal insulation as needed. Successful induction of ischemic stroke was confirmed histologically. No additional postoperative analgesics were administered beyond the anesthetic regimen described above. Mice were sacrificed 7 days after tMCAO, and brains were fixed in 4% PFA. Coronal brain sections (40 µm) were prepared using a cryostat (Leica). All procedures were performed in a randomized manner, and investigators were blinded to group allocation during subsequent analyses.

### 2.4. Immunostaining and Image Acquisition

Cryosections were rehydrated with PBS, then permeabilized and blocked with 5% goat serum in PBS with 0.3% TritonX-100. Following overnight incubation with primary antibodies at 4 °C, sections were washed, incubated with secondary antibodies at RT for 2 h, and counterstained with Hoechst33342 (Invitrogen Molecular Probes, Eugene, OR, USA) before being mounted with 90% glycerol containing 2% propyl gallate for observation under the microscope. Primary antibodies used are as follows: Anti-AKH-C′ (1:1000, Generated antibody), Anti-AKH-LCCL (1:1000, Generated antibody), Anti-Iba1 (1:1000, Fuji film, Tokyo, Japan #019-19741), Anti-MMR/CD206 (1:500, R&D systems, Minneapolis, MN, USA #AF2535), Anti-P2RY12 (1:200, Novus Biologicals, Centennial, CO, USA #NBP1-78249), Anti-PH3 (1:100, Invitrogen, Carlsbad, CA, USA #MA5-15220), Anti-SOX2 (1:200, Abcam, Cambridge, UK #ab92494), Anti-Ki67 (1:1000, BD Pharmingen, San Diego, CA, USA #556003), Anti-Flk1 (1:50, BD Pharmingen #550549), Anti-s100β (1:100, Abcam #ab52642), Anti-Tbr1 (1:200, Abcam #ab31940), Anti-BrdU (5-bromo-2′-deoxyuridine) (1:250, Abcam #ab6326), Anti-GFAP (1:200, Thermo Fisher Scientific, Waltham, MA, USA #13-0300), Anti-Tuj1 (β-III-tubulin) (1:300, R&D systems #MAB1195), Anti-NG2 (1:100, Abcam #ab5320), Anti-Caspase3 (1:500, Cell Signaling Technology, Danvers, MA, USA #9664), Anti-ZO1 (1:10, Invitrogen #40-2200), Claudin-2 (1:400, Curr. Biol), Anti-Vcam1 (1:1000, Millipore™, Burlington, MA, USA #CBL1300), Anti-Acetylate tublin (1:1000, Sigma-Aldrich, St. Louis, MO, USA #T 6793), Anti- IB4 conjugate antibody (1:300, Invitrogen #MP 21410), Anti-CD68 (1:300, Bio-Rad Laboratories, Hercules, CA, USA #FA-11). For imaging of neurospheres and immunohistochemistry (IHC) samples, images were acquired using a TCS SP8 STEDmicroscope (Leica) or an BX53 microscope (Olympus, Tokyo, Japan).

### 2.5. BrdU Labeling

For analysis of proliferating cells, 5-bromo-2′-deoxyuridine (BrdU; 50 mg/kg body weight) was administered by intraperitoneal injection. For embryonic analysis, BrdU was injected into pregnant dams at embryonic day (E) 16.5, and fetal brains were collected 30 min after injection. Following BrdU administration, mice were sacrificed, and brains were immediately dissected, fixed, cryoprotected, and sectioned as described above. For BrdU antigen retrieval, brain sections were treated with boiling sodium citrate buffer, followed by a brief wash in cold 1× PBS. Subsequent immunohistochemical procedures were performed as described in the IHC section.

### 2.6. Neurosphere Culture and In Vitro Differentiation

Neurospheres culture procedures were described in [15,16]. In brief, a fine thin layer of SVZ from LV wall and whole hippocampus were dissected carefully from P5 mouse brain. Samples were collected in calcium–magnesium-free phosphate buffer (CMF) and then digested with disperse II at 37 °C for 10 min. Then, trypsin/ethylenediaminetetraacetic acid (Trypsin-EDTA) (Sigma-Aldrich) was applied for 5 min at 37 °C. Trypsin-EDTA was neutralized by trypsin neutralizer (Gibco^®^, Thermo Fisher Scientific, Waltham, MA, USA) by adding double volume of the Trypsin-EDTA applied. Then, sample tissues were fragmented by pipetting and saturated with the culture medium (B27 supplement, 20 µg/mL epidermal growth factor (EGF), 20 µg/mL fibroblast growth factor (FGF), 2 mM glutamine and heparin in dulbecco’s modified eagle medium (DMEM)). For primary culture, cells were plated at 5 × 10^4^ cells/well density in a 24-well plate previously coated with poly 2-hydroxyethyl methacrylate (20 mg/mL; Sigma) and cultured for 7 days. To obtain secondary neurospheres, passage transfer was performed from primary neurospheres following the procedures above except disperse II treatment, and cells were seeded at 1 × 10^3^ cells/well density in a 96-well plate and cultured for another 7 days. For in vitro differentiation, three secondary neurospheres for each sample were put onto the coated surface of coverslip submerged in the differentiation induction medium (0.5× B27 supplement, 1 mM glutamine and 1% fetal calf serum in DMEM). For coating, a mixture (1:1) of poly-L-ornithine (Sigma, Life Science) and laminin (Sigma, L2020) was used and coated coverslips were incubated at 37 °C and 5% CO_2_ overnight. After 7 days of culture, IHC was performed on the monolayer of differentiated cells generated from neurospheres following the procedures mentioned in the earlier section.

### 2.7. Production of Anti-AKH Antibody

Two polyclonal antibodies against mouse AKH were generated in rabbits (Hokudo Co., Ltd., Sapporo, Japan). For the C-terminal–specific antibody, a synthetic peptide corresponding to the C-terminal region of mouse AKH (N′–APRII QNICT EFNSQ PRN–C′) were conjugated to keyhole limpet hemocyanin (KLH) using a carbodiimide-mediated coupling reaction according to the manufacturer’s protocol and used for immunization. For the LCCL domain–specific antibody, synthetic peptides corresponding to the LCCL domain of AKH (N′–HSGVL DNSGG KILVR KVACQ–C′) were similarly conjugated to KLH. Rabbits were immunized subcutaneously four times at two-week intervals with peptide–Freund’s adjuvant emulsions. Serum was collected one week after the final boost, and IgG fractions were purified using a protein-A affinity column (GE Healthcare, Tokyo, Japan). The specificity of each antibody was verified by IHC and Western blotting using mouse brain tissues, and by pre-absorption with the respective immunizing peptides. The purified antibodies were stored at −80 °C in PBS containing 50% glycerol.

### 2.8. Production of AKH-LCCL Reverse Peptide

A synthetic peptide corresponding to the reverse sequence of the AKH LCCL domain (N′–QGAVK RVLIK GGSND LVGSH C–C′) was custom synthesized at >95% purity (Hokudo Co., Ltd). Lyophilized peptides were dissolved in sterile PBS to prepare stock solutions (1 mg/mL) and stored at −80 °C until use. Working solutions were freshly prepared at a final concentration of 1 µg/mL in sterile PBS prior to each experiment. For bacterial colony formation assays, the peptide solution was mixed with warm 0.5% agarose medium and poured into culture dishes. After solidification, bacteria were inoculated and cultured at 37 °C for 16–18 h. To evaluate the effects of the peptide on cell attachment and morphology, bacteria were also cultured on glass slides pre-coated with the peptide at the same concentration (1 µg/mL). Slides were placed in a humidified chamber to prevent drying during incubation. After incubation, samples were processed for subsequent immunostaining and microscopic analysis.

### 2.9. Embryonic Cerebrospinal Fluid Collection

CSF was collected by inserting a glass capillary into cisterna magna, centrifuged 1000× *g* for 10 min at 4 °C to remove any cellular debris, and the supernatant was stored at −80 °C until use. Samples contaminated with blood were excluded. This was processed as described previously [19].

### 2.10. Cytokine Profiling of CSF

CSF samples were analyzed using the RayBio^®^ C-Series Mouse Cytokine Antibody Array (RayBiotech, Inc., Peachtree Corners, GA, USA) according to the manufacturer’s instructions. Briefly, each membrane pre-spotted with capture antibodies against mouse cytokines were first blocked with blocking buffer provided in the kit for 30 min at RT with gentle rocking. CSF from AKH^+^/^+^ and AKH^−^/^−^ was diluted 1:2 with the array buffer in the kit. After blocking, diluted CSF samples were transferred to the membranes and incubated overnight at 4 °C gentle rocking to allow cytokines in the samples to bind to their corresponding capture antibodies immobilized on the membrane. Following sample incubation, membranes were washed three times thoroughly with the wash buffer provided in the kit to remove unbound proteins. Membranes were then incubated with a cocktail of biotinylated detection antibodies for 2 h at RT. After another series of washes, the membranes were incubated with HRP-conjugated streptavidin for 2 h at RT.

A signal was detected using chemiluminescent substrate (ECL, Thermo Fisher Scientific) and imaged using a chemiluminescence imaging system (Image Quant LAS 4000, GE Healthcare). Each cytokine array experiment was performed twice using independent CSF samples. For each cytokine, two duplicate spots were present per membrane; therefore, signal intensities from a total of four spots (two spots per membrane–two independent experiments) were averaged and used for subsequent quantitative analysis. Spot intensities were quantified using Fiji (ImageJ, version 2.9.0; National Institutes of Health, Bethesda, MD, USA). Signal intensities were normalized to the positive controls on each membrane, and background signals were subtracted using local background correction.

### 2.11. Western Blotting

For tissue Western blotting, Ganglionic Eminence (GE) was collected from E14.5 mouse brain then suspended with PBS immediately and lysed in a 2× sample buffer. For CSF sampling, CSF was collected by inserting a glass capillary into cisterna magna, then centrifuged 10,000× *g* for 10 min. This processed as described [19]. CSF samples were diluted four-fold and mixed with sample buffer. GE tissues were lysed in RIPA buffer (50 mM Tris-HCl pH 7.4, 150 mM NaCl, 1% NP-40, 0.1% SDS, 0.5% sodium deoxycholate) supplemented with protease and phosphatase inhibitor cocktails (Nacalai Tesque or Sigma-Aldrich). Lysates were incubated on ice for 30 min and centrifuged at 15,000× *g* for 15 min at 4 °C. Equal amounts of protein (20 μg) were mixed with 2× SDS sample buffer (250 mM Tris-HCl pH 6.8, 8% SDS, 40% glycerol, 0.02% bromophenol blue, 10% β-mercaptoethanol) and boiled for 5 min at 95 °C. Samples were separated by SDS-PAGE using 14% polyacrylamide gels and transferred to PVDF membranes (Millipore, Burlington, MA, USA) using a semi-dry transfer system. Membranes were blocked in 10% NDS in TBS-T buffer (20 mM Tris-HCl pH 7.6, 150 mM NaCl, 0.1% Tween-20) for 30 min at RT. Membranes were then incubated overnight at 4 °C with primary antibodies diluted in blocking buffer. After washing with TBS-T, membranes were incubated with HRP-conjugated secondary antibodies for 1 h at RT. Signal was detected using an enhanced chemiluminescence substrate, and images were captured using a chemiluminescence imaging system (Image Quant LAS 4000, GE Healthcare, Tokyo, Japan). Band intensities were quantified using ImageJ (NIH) and normalized to a housekeeping protein GAPDH.

### 2.12. Maternal Immune Activation

For timed pregnancies, females were checked for the plugs, and the date of the plug was noted as embryonic day 0.5 (E0.5). Bodyweight was monitored every 3 days. On E12.5, pregnant dams received a single dose (20mg/kg, i.p.) of polyinosinic-polycytidylic acid (poly(i:c), Sigma Aldrich) or saline as vehicle control.

### 2.13. RNA Extraction and qRT-PCR

Total RNA was extracted from GE of E14.5 moue brain and cultured neurospheres was isolated and purified by using the RNeasy^®^ Mini Kit (QIAGEN, Hilden, Germany, Cat No. 74106), according to the manufacturer’s instructions. RNA concentration and purity were assessed spectrophotometrically by measuring the A260/A280 ratio, and 1 µg of total RNA was reverse transcribed into cDNA using the SuperScript™ II reverse transcriptase kit (Invitrogen, Cat. No. 18064022). Quantitative PCR analysis was performed using Luna^®^ universal qPCR master mix (New England BioLabs, Ipswich, MA, USA, Cat. No. M3003E) on a Step Oneplus Real-Time PCR System (Applied Biosystems, Foster City, CA, USA). The thermal cycling protocol consisted of 95 °C for 1 min, followed by 40 cycles of 95 °C for 15 seconds (s) and 60 °C for 30 s. Relative gene expression levels were calculated using the ΔΔCt method, with GAPDH serving as the internal control. The following primer sequences were used:

Hes1, forward 5′-AAAGCCTATCATGGAGAAGAGGCG-3′ and reverse 5′-GGAATGCCGGGAGCTATCTTTCTT-3′;
Hes5, forward 5′-AGAAAAACCGACTGCGGAAGCC-3′ and reverse 5′-CGCGGCGAAGGCTTTGCT-3′;
IL-1r, forward 5′-GCCTCGTGTCGGACCCAT-3′ and reverse 5′-TCCTTTGAGCCCAAGGCCAC-3′;
Cxcl16, forward 5′-TCCTTTTCTTGTTGGCGCTG-3′ and reverse 5′-CAGCGACACTGCCCCTGGT-3′;
ADAM TS4 forward 5′-CAGGTCCCATGTGCACGT-3′ and reverse 5′-CATCTGCCACCACCAGTGTCT-3′;
Tnfa, forward 5′-TCCCAGGTTCTCTCAAGGGA-3′ and reverse 5′-GGTGAGGAGCACGTAGTCGG-3′;
VEGF-r(2), forward 5′-TCACCGAGAACAAGAACAAA-3′ and reverse 5′-TCCTATATCCTACAACCACAA-3′;
IL-6, forward 5′-AAAGAGTTGTGCAATGGCAATTCT-3′ and reverse 5′-AAGTGCATCATCGTTGTTCATACA-3′;
GAPDH, forward 5′-AGCTTGTCATCAACGGGAAG-3′ and reverse 5′-TTTGATGTTAGTGGGGTCTCG-3′.

### 2.14. Bacterial Culture with Peptides

Bacterial cultures were prepared using *Escherichia coli* DH5a. Overnight cultures were grown in LB medium at 37 °C with shaking at 200× *g*. The cultures were diluted to an optical density at 600 nm (OD600) of 0.1 and incubated with synthetic AKH-LCCL peptide and AKH-LCCL reverse peptides at 1 µg/mL. For colony formation assays, bacterial suspensions were plated on LB agar plates containing the peptide and incubated overnight at 37 °C. Colony-forming units were counted to assess bacterial viability. For immunostaining assays, bacterial suspensions were placed on slide glasses and incubated in a humidified chamber to prevent drying. After incubation with AKH-LCCL peptide, slides were processed for immunofluorescence staining according to standard protocols. All experiments were performed in triplicate, and representative images or counts were used for analysis.

### 2.15. CSF Label-Free Quantitative Proteomics Analysis

Label-free quantitative proteomic analysis of mouse CSF samples was performed by filgen Inc. (Nagoya, Japan). Two independent CSF samples (AKH^+^/^+^ and AKH^−^/^−^) were subjected to tryptic digestion and analyzed using a nano LC-MS/MS platform. Protein identification and label-free quantification were performed based on the intensity of MS1 peptide ion signals.

#### 2.15.1. Chemicals and Instrumentation

DL-dithiothreitol (DTT), iodoacetamide (IAA), formic acid (FA), and acetonitrile (ACN) were purchased from Sigma (St. Louis, MO, USA). Trypsin from bovine pancreas was purchased from Promega (Madison, WI, USA). Ultrapure water was prepared from a Millipore purification system (Billerica, MA, USA). An Ultimate 3000 nano UHPLC system coupled with a Q Exactive HF mass spectrometer (Thermo Fisher Scientific, USA) with an ESI nanospray source.

#### 2.15.2. SDS-PAGE

Protein samples were separated by SDS–polyacrylamide gel electrophoresis (SDS-PAGE) using a 12% separating gel. Electrophoresis was performed at 80 V for 20 min, followed by separation at 120 V for 1 h. After electrophoresis, gels were subjected to silver staining using a commercially available silver staining kit (Wako, Osaka, Japan) according to the manufacturer’s instructions.

#### 2.15.3. In-Gel Digestion

Protein bands were excised from silver-stained gels and cut into approximately 1 mm^3^ pieces. Gel pieces were destained using a solution containing potassium ferricyanide and sodium thiosulfate, followed by washing with water and ammonium bicarbonate. The gel pieces were dehydrated with acetonitrile and subsequently reduced with dithiothreitol (DTT) at 56 °C, then alkylated with iodoacetamide (IAA) at RT in the dark. After additional dehydration with acetonitrile, proteins were digested with trypsin overnight at 37 °C. Peptides were extracted from the gel pieces using ammonium bicarbonate/acetonitrile solution, combined, and lyophilized to near dryness. The resulting peptides were resuspended in 0.1% formic acid prior to LC–MS/MS analysis.

#### 2.15.4. Nano LC-MS/MS Analysis

##### NanoLC

Peptide samples were analyzed using a nanoflow UPLC system (Ultimate 3000 nano UHPLC, Thermo Fisher Scientific, USA) equipped with a trapping column (PepMap C18, 100 Å, 100 μm × 2 cm, 5 μm) and an analytical column (PepMap C18, 100 Å, 75 μm × 50 cm, 2 μm). Approximately 1 μg of peptides was loaded onto the system. The mobile phases consisted of 0.1% formic acid in water (buffer A) and 0.1% formic acid in 80% acetonitrile (buffer B). Peptides were separated at a flow rate of 250 nL/min using a linear gradient of buffer B from 2% to 8% over 3 min, from 8% to 20% over 56 min, from 20% to 40% over 37 min, and finally from 40% to 90% over 4 min.

##### Mass Spectrometry

Mass spectrometric analysis was performed in data-dependent acquisition mode. Full MS scans were acquired over an *m*/*z* range of 300–1650 at a resolution of 60,000 (at *m*/*z* 200), with an automatic gain control (AGC) target of 3 × 10^6^. MS/MS analysis was conducted using a Top 20 method at a resolution of 15,000 (at *m*/*z* 200), with an AGC target of 1 × 10^5^ and a maximum injection time of 19 ms. Peptide fragmentation was achieved by higher-energy collisional dissociation with a normalized collision energy of 28%. The isolation window was set to 1.4 Th, and precursor ions with unassigned charge states, singly charged ions, or charge states greater than 6 were excluded. Dynamic exclusion was enabled with a duration of 30 s.

#### 2.15.5. Data Analysis

Raw MS data were processed and analyzed using MaxQuant software (version 1.6.2.6) and searched against a mouse protein database appropriate for the sample species. Carbamidomethylation of cysteine residues was set as a fixed modification, and oxidation of methionine was specified as a variable modification. Trypsin was selected as the proteolytic enzyme, allowing up to two missed cleavages. The precursor mass tolerance was set to 10 ppm, and the fragment ion mass tolerance was set to 0.6 Da.

### 2.16. Behavior Test Batch

All behavioral experiments were conducted during the light period of the light/dark cycle. Although mice are nocturnal animals, behavioral assessments conducted during the light phase are widely used and allow reliable detection of genotype-dependent differences when all experimental groups are tested under identical conditions. To minimize stress-related variability, mice were habituated to handling for at least three days prior to behavioral testing.

#### 2.16.1. Open Field Test

Open field test was conducted as previously described [20]. Each mouse was placed in the center of the open field apparatus (40 cm × 40 cm × 30 cm; Accuscan Instruments, Columbus, OH, USA). Total distance traveled (in cm), vertical activity (rearing measured by counting the number of photobeam interruptions), time spent in the center, and the beam-break counts for stereotyped behaviors were recorded. Data was collected for 120 min.

#### 2.16.2. Elevated Plus Maze Test

Elevated plus maze test was conducted as previously described [21]. The elevated plus maze (O’Hara & Co., Tokyo, Japan) consisted of two open arms (25 cm × 5 cm) and two enclosed arms of the same size, with 15 cm high transparent walls. The arms and central square were made of white plastic plates and were elevated to a height of 55 cm above the floor. To minimize the likelihood of animals falling from the apparatus, 3 mm high plastic ledges were provided for the open arms. Arms of the same type were arranged on opposite sides to each other. Each mouse was placed in the central square of the maze (5 cm × 5 cm), facing one of the closed arms. The level of lighting in the room was 100 lux. Mouse behavior was recorded during a 10 min test period. The number of entries, and the time spent in the open and enclosed arms, were recorded. For data analysis, we used the following four measures: the percentage of entries into the open arms, the time spent in the open arms (s), the number of total entries, and total distance traveled (cm). Data acquisition and analysis were performed automatically using Smart 3.0 video tracking system (Panlab, Harvard Apparatus, Holliston, MA, USA).

#### 2.16.3. Light/Dark Transition Test

Light/dark transition test was conducted as previously described [20]. The apparatus used for the light/dark transition test consisted of a cage (40 cm × 40 cm × 30 cm) divided into two sections of equal size by a partition containing a door (Accuscan Instruments, Columbus, OH, USA). One chamber was brightly illuminated (200 lux), whereas the other chamber was dark (2 lux). Mice were placed into the dark side and allowed to move freely between the two chambers with the door open for 10 min. The total number of transitions between chambers, time spent in each side, first latency to enter the light side and distance traveled were recorded automatically.

#### 2.16.4. Rotarod Test

The rotarod test, using an accelerating rotarod (UGO Basile Accelerating Rotarod), was performed by placing mice on rotating drums (3 cm diameter) and measuring the time each animal was able to maintain its balance on the rod. The speed of the rotarod accelerated from 5 to 40 rpm over a 5 min period.

#### 2.16.5. Environment Exposures and Contextual Fear Conditioning

Contextual fear conditioning and memory retrieval tests were performed using the TimeFZ4 system (O’Hara & Co., Ltd., Tokyo, Japan), following the protocol previously described [21]. The protocol consisted of a training session followed by a context and cue test on the following day. During training, mice were placed in a conditioning chamber (33 × 25 × 28 cm, 150 lux) and allowed to explore for 3 min, followed by a 30 s tone (conditioned stimulus, CS; white noise, 55 dB), which co-terminated with a 2 s foot shock (unconditioned stimulus, US; 0.75 mA). This CS–US pairing was repeated three times with 1 min inter-trial intervals. Twenty-four hours later, mice were returned to the same chamber for a 5 min context test (no tone or shock). Two hours after the context test, mice were placed in a novel chamber with different floor texture and odor and exposed to the tone alone (cue test). Freezing behavior (immobility except for respiration) was measured and scored using automated methods.

### 2.17. Statistics

Animals were randomly assigned to experimental groups. Investigators performing data acquisition and quantitative analyses were blinded to genotype and experimental conditions until completion of the analyses. Quantitative data were shown as mean ± standard deviation (SD). Unpaired two-tailed Student’s *t*-tests were used for comparisons between two groups. For experiments involving three or more groups, statistical significance was assessed using one-way ANOVA. Significance was set as follows: * *p* < 0.05; ** *p* < 0.01, *** *p* < 0.001.

## 3. Results

### 3.1. AKH Is Expressed on the Apical Surface of NSCs Facing the Ventricles and Is Secreted into the ChP

To determine the spatial distribution of AKH protein during early brain development, we examined the mouse brains at E14.5 using a newly generated AKH-specific antibody. The specificity of the anti-Akhirin antibody was validated by immunofluorescence analysis, as shown in Supplementary Figure S1. At this developmental stage, NSCs exhibit high proliferative activity, enabling detailed analysis of protein localization along the lateral ventricle (LV).

**Figure 1:**
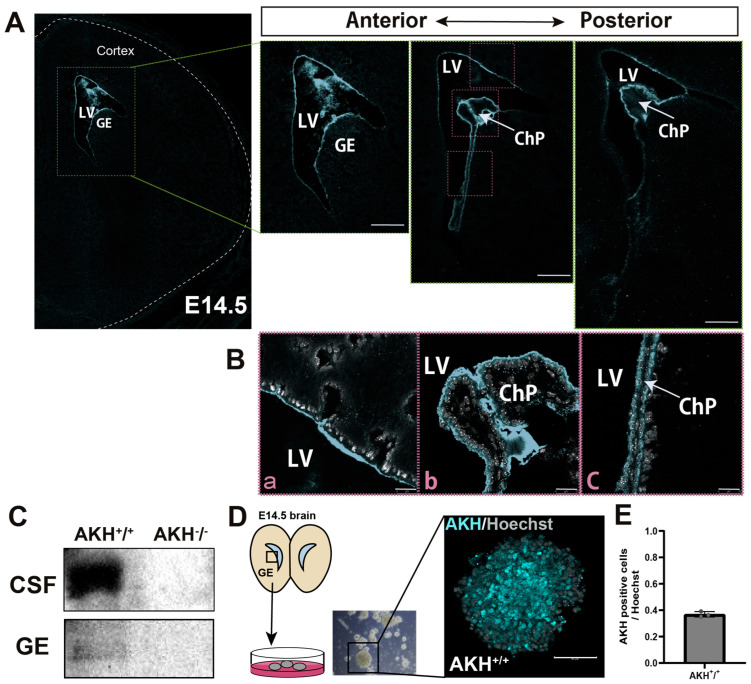
The expression of AKH at the E14.5 mice brain. (**A**) Immunohistochemistry shows AKH expression in the LV extending from anterior to posterior regions of the E14.5 mouse brain. Scale bar: 1 mm (whole brain) and 100 µm (anterior–posterior images). (**B**) High magnification images showing AKH immunoreactivity on the apical surface (**a**), ChP (**b**) and the lower part of the ChP (**c**) from the middle image of (**A**). Scale bars: 25 µm. (**C**) Western blot analysis showing AKH expression in CSF and GE at E14.5. (**D**) Immunocytochemical analysis showing AKH expression in secondary neurosphere. (**E**) Quantification of AKH-positive cells, normalized to the total number of Hoechst-positive nuclei, in sections of neurospheres derived from the GE at E14.5 (n = 3 independent neurosphere cultures). Data are presented as mean ± SD. Scale bars:50 µm. LV: Lateral ventricle. GE: Ganglionic Eminence. ChP: Choroid Plexus. CSF: Cerebrospinal fluid. SVZ: Subventricular zone.

IHC analysis revealed that AKH is expressed in the periventricular region, with expression extending from the anterior to posterior ventricular regions (Figure 1A). At higher magnification, AKH immunoreactivity was detected on the apical surface of NSCs facing the ventricular lumen (Figure 1(Ba)). In addition, strong AKH expressions were observed on the surface of ChP (Figure 1(Bb,Bc)), a tissue responsible for CSF secretion into the ventricles. Furthermore, the expression of AKH in the CSF at E14.5 was confirmed by Western blotting (Figure 1C, Supplementary Figure S2A,B). In parallel, we harvested GE regions from E14.5 brains and performed Western blotting which revealed detectable AKH expression (Figure 1C, Supplementary Figure S2C,D). The GE tissue was subsequently subjected to neurospheres formation assays. Immunostaining of the generated neurospheres showed mosaic expression of AKH (Figure 1D). Quantification of AKH immunoreactivity in neurosphere-forming cells showed that approximately 37% of the cells were AKH-positive (Figure 1E).

Taken together, these results indicate that AKH is enriched at the apical surface of the VZ and ChP and is secreted into the CSF at E14.5. Given that NSCs are arranged along the ventricular surface and that CSF components directly influence their proliferation and differentiation during early development, these findings suggest that AKH may contribute to the regulation of NSCs proliferation at this stage.

### 3.2. Loss of AKH Disrupts NSC Niche Homeostasis, Including NSCs Proliferation

**Figure 2:**
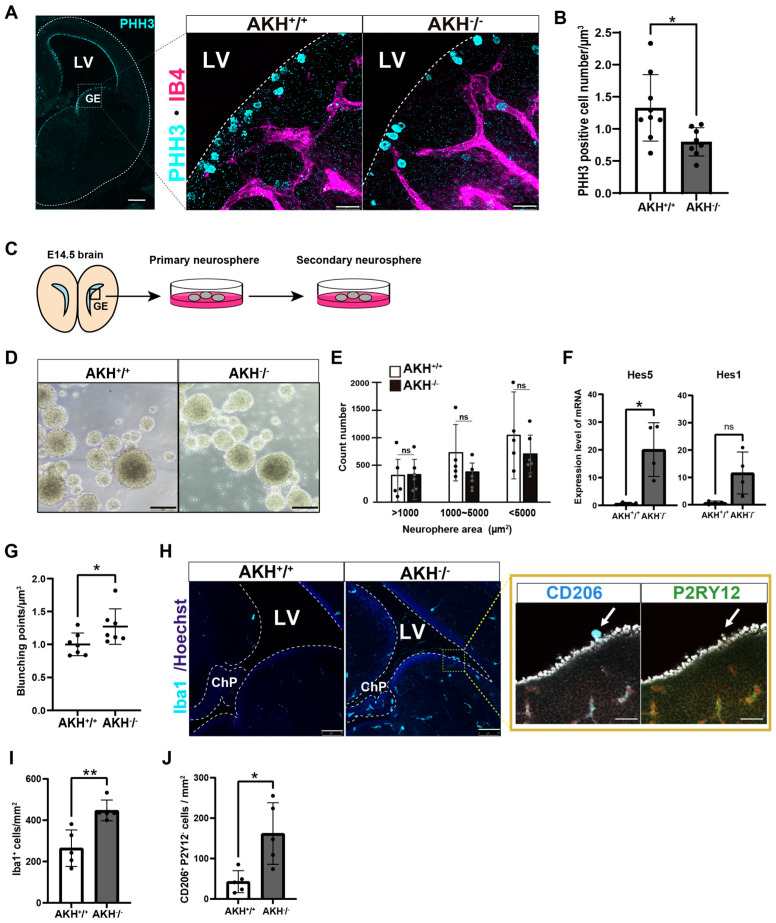
Loss of AKH disrupts neurogenic niche homeostasis and reduces NSCs proliferation in vivo. (**A**) Immunofluorescence images of the LV region in E14.5 mouse brains showing PHH3^+^ proliferative NSCs (cyan) and IB4-labeled blood vessel (magenta). Scale bars:1 mm and 25 µm. The dashed line indicates the boundary of the LV. (**B**) Quantification of PHH3^+^ cells on the ventricular apical surface of AKH^+^/^+^ and AKH^−^/^−^ brains using three-dimensional images (*Z*-axis depth, 70 µm). AKH^+^/^+^: n = 9, AKH^−^/^−^: n = 8. *p* = 0.0463 (**C**) Schematic illustration of the neurosphere assay. (**D**) Representative image of secondary neurosphere from AKH^+^/^+^ and AKH^−^/^−^. Scale bars:75 µm. (**E**) Quantification of neurosphere area (µm^2^) measured by ImageJ. AKH^+^/^+^: n = 5, AKH^−^/^−^: n = 6. area > 1000: *p* = 0.943, area 1000–5000: *p* = 0.184, <5000: *p* = 0.408. (**F**) Relative mRNA expression levels of *Hes5* and *Hes1* in GE tissue. AKH^+^/^+^: n = 4, AKH^−^/^−^: n = 4. Hes5: *p* = 0.0374, Hes1: *p* = 0642. (**G**) Quantification of blood vessels branching points using three-dimensional images (*Z*-axis depth, 70 µm) with Imaris ver9.5 (µm^3^). *p* = 0.0492. (**H**) Immunofluorescence images of the LV region in E14.5 mouse brains showing Iba1^+^ microglia (cyan), CD206^+^ (cyan) and P2RY12^+^ (green). Scale bars:75 µm and 25 µm. The dashed line indicates the boundary of the LV. (**I**,**J**) Quantification of Iba1^+^ and CD206^+^ P2RY12^-^ cell densities in the LV of AKH^+^/^+^ and AKH^−^/^−^ embryos. AKH^+^/^+^: n = 5, AKH^−^/^−^: n = 5. (**I**) *p* = 0.0061, (**J**) *p* = 0.0385 Data are presented as mean ± SD; * *p* < 0.05, ** *p* < 0.01 (Student’s *t*-test). ns, not significant.

To investigate the role of AKH in regulating NSCs/NPCs proliferation during early brain development, we analyzed the proliferative status of NSCs/NPCs in AKH-deficient (AKH^−^/^−^) embryos at E14.5. Immunostaining for phosphorylated histone H3 (PHH3), a marker of mitotic cells, revealed a significant reduction in proliferating NSCs/NPCs in AKH^−^/^−^ embryos compared to AKH^+^/^+^ embryos (Figure 2A). PHH3-positive cells were specifically quantified among apical NSCs lining the ventricular surface, and their density was compared between AKH^+^/^+^ and AKH^−^/^−^ embryonic mice (Figure 2B). Similarly, when the ventricular surface was observed from the ventricular side, Hoechst staining revealed an increased proportion of NSCs/NPCs in interphase, indicating that fewer cells were undergoing mitosis (Supplementary Figure S2E,F). In contrast, in vitro neurospheres formation assays using cells from the GE of E14.5 embryos revealed no significant differences in the number or size of secondary neurospheres between AKH^−^/^−^ and AKH^+^/^+^ embryos after two weeks of culture (Figure 2C–E). Quantitative RT-qPCR analysis showed that the expression levels of stemness-associated transcription factors, Hes1 and Hes5, were upregulated in NSCs/NPCs isolated from GE tissue of AKH^−^/^−^ at E14.5 (Figure 2F). These findings suggest that AKH-deficient NSCs/NPCs tend to maintain an undifferentiated state and that their intrinsic proliferative capacity is preserved under controlled in vitro conditions.

Considering previous reports describing the close association between NSCs/NPCs and the vasculature within the neurogenic niche [22,23], we next investigated whether vascular architecture was altered in AKH^−^/^−^ embryos. Quantitative analysis revealed a significant increase in vascular bifurcation points in the AKH^−^/^−^ cortex, without changes in total vascular area or volume, suggesting aberrant vessel branching and remodeling (Figure 2G, Supplementary Figure S2G,H). These morphological alterations may reflect a disrupted niche microenvironment that contributes to impaired NSCs proliferation in vivo. Given that embryonic angiogenesis and immune cell recruitment are tightly coordinated during brain development [24,25], we next examined the number and distribution of Iba1^+^ immune cells. We observed a significant increase in Iba1^+^ cells in the VZ of AKH^−^/^−^ embryos compared to AKH^+^/^+^ embryos (Figure 2H), indicating excessive recruitment or retention of macrophage-lineage cells within the neurogenic niche. To distinguish between microglia and ChP-derived macrophages, we performed co-staining for CD206 (a ChP macrophage marker) and P2RY12 (a microglia-specific marker). The majority of Iba1^+^ cells at the ventricular surface of AKH^−^/^−^ brains were CD206^+^/P2RY12^−^, suggesting infiltration of ChP-derived macrophages into the neurogenic niche (Figure 2I,J). The abnormal presence of these immune cells may further destabilize the NSC/NPC microenvironment and exacerbate defects in proliferation and differentiation.

Taken together, these findings demonstrate that AKH is required for maintaining a neurogenic niche that supports proper NSCs proliferation and fate specification in vivo. Loss of *AKH* disrupts the balance between self-renewal and differentiation, likely through alterations in the local microenvironment rather than cell-intrinsic mechanisms.

### 3.3. AKH Deficiency Disrupts the Timing of Differentiation into Neurons and Glial Cells

Given that AKH deficiency attenuates NSCs proliferation during mid-embryonic development, we next investigated whether it also affects subsequent cortical development and glial differentiation.

**Figure 3:**
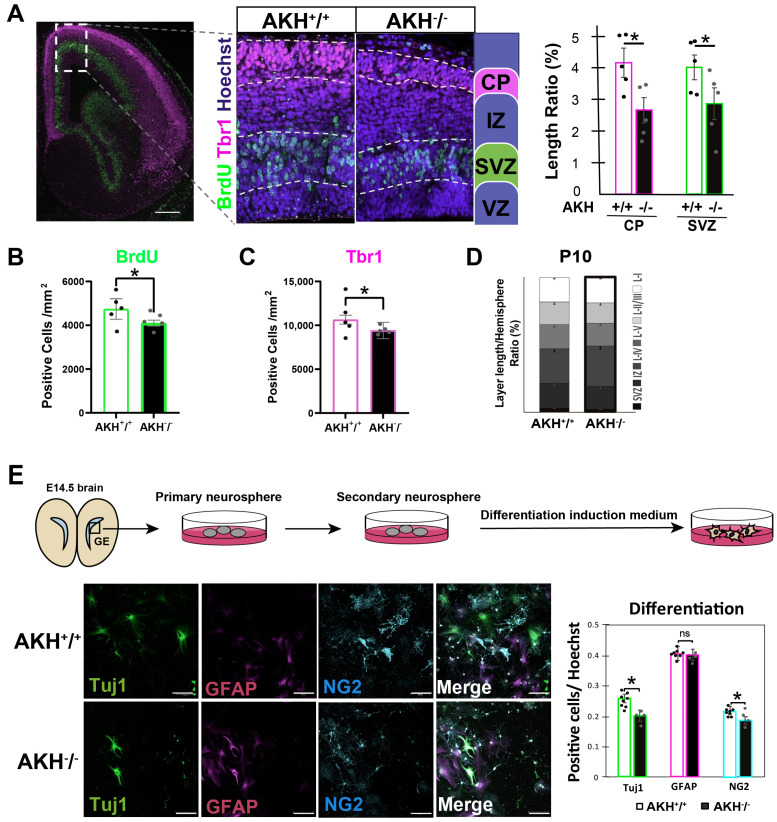
Loss of AKH delays neuronal differentiation and accelerates glial differentiation during cortical development. (**A**) Immunofluorescence images of cortical layer in E16.5 mouse brain showing BrdU^+^, Tbr1^+^, and Hoechst staining. Layer thickness was quantified and normalized to the total length of the cerebral hemisphere. Scale bar:1mm. AKH^+^/^+^: n = 5, AKH^−^/^−^: n = 5. CP: *p* = 0.0180, SVZ: *p* = 0.0367. (**B**,**C**) Quantification of BrdU^+^ or Tbr1^+^ cells at E16.5. AKH^+^/^+^: n = 5, AKH^−^/^−^: n = 5. (**D**) Quantification of cortical layer thickness normalized to the total length of the cerebral hemisphere at P10. AKH^+^/^+^: n = 5, AKH^−^/^−^: n = 5. (**E**) Schematic illustration of neurosphere culture and induced differentiation. Quantification of marker-positive cells normalized to the total number of Hoechst^+^ cells. Scale bars:100 µm. AKH^+^/^+^: n = 8, AKH^−^/^−^: n = 4. Tuj1: *p* = 0.0445, NG2: *p* = 0.0457. Data are presented as mean ± SD; * *p* < 0.05, (Student’s *t*-test). ns, not significant.

We hypothesized that reduced NSCs proliferation in AKH^−^/^−^ embryos influences cortical layer formation and neuronal differentiation. To assess proliferative activity during late corticogenesis, 5-bromo-2′-deoxyuridine (BrdU) was intraperitoneally injected into pregnant mice at E 16.5, and fetal brains were collected 30 min later. Because short BrdU pulses label only a subset of cells undergoing S-phase, BrdU incorporation was used as an indicator of ongoing proliferation rather than a marker of specific anatomical compartments. The thickness and cellular density of BrdU-positive cells and the Tbr1-positive cortical plate (CP) were assessed at E16.5. The relative length of the SVZ and CP were quantified by normalizing to the total length of the cerebral hemisphere from the parietal to the basal plane. This analysis revealed that both the BrdU-positive layers and the Tbr1-positive cortical plates were thinner in AKH^−^/^−^ compared with AKH^+^/^+^ controls (Figure 3A). Quantification showed a significant reduction in the density of BrdU^+^ cells within the SVZ of AKH^−^/^−^ (Figure 3B). Similarly, the density of Tbr1^+^ neurons was significantly reduced in the cortical plate of AKH^−^/^−^ embryos (Figure 3C), indicating delayed neuronal differentiation and impaired cortical development. By P10, when the cortical layers are largely established, no significant differences in cortical layer thickness or organization were observed between AKH^−^/^−^ and AKH^+^/^+^ mice (Figure 3D), suggesting that the developmental delay observed during embryogenesis is largely compensated during postnatal cortical maturation.

To further investigate whether AKH deficiency affects the differentiation potential of NSCs, we examined neurosphere-derived differentiation in vitro. Neurospheres were cultured for 21 days in a differentiation-inducing medium to generate monolayers containing neurons and glial cells. Cells were immunolabeled using three major cell lineage markers; immature neuronal marker Tuj1, astrocyte marker GFAP, and oligodendrocyte precursor cells marker NG2 (Figure 3E). This analysis revealed a significant reduction in the number of Tuj1- and NG2-expressing cells (Figure 3E), indicating impaired neuronal and oligodendroglial differentiation.

These findings suggest that the differentiation potential of NSCs/NPCs is altered under AKH-deficient conditions. This observation is partially consistent with our previous reports using neurospheres from P5 mice [15]. While we previously reported that gliogenesis was not impaired in the AKH^−^/^−^ neurogenic niche at postnatal stages [15], the present analysis using E14.5-derived neurospheres indicates that both oligodendrogenesis and neurogenesis are affected. These results suggest that AKH contributes to temporal regulation of NSC/NPC fate decisions, particularly during the transition from embryonic to early postnatal development. Consistent with this interpretation, neurospheres derived from AKH^−^/^−^ embryos maintained elevated expression of Hes1 and Hes5, transcription factors associated with NSC stemness maintenance (Figure 2F), suggesting that AKH deficiency prolongs the undifferentiated state and delays neuronal and glial differentiation.

### 3.4. AKH Deficiency Disrupts the Barrier Function of the ChP and Alters the Composition of the CSF

Although the intrinsic proliferative capacity of NSCs appeared intact under defined culture conditions, the marked reduction in proliferation observed in vivo at E14.5 suggested the involvement of inhibitory extrinsic factors within the AKH^−^/^−^ neurogenic niche. Given that the CSF is in direct contact with the apical surface of NSCs during early brain development, we hypothesized that alterations in CSF composition contribute to NSCs dysfunction.

**Figure 4:**
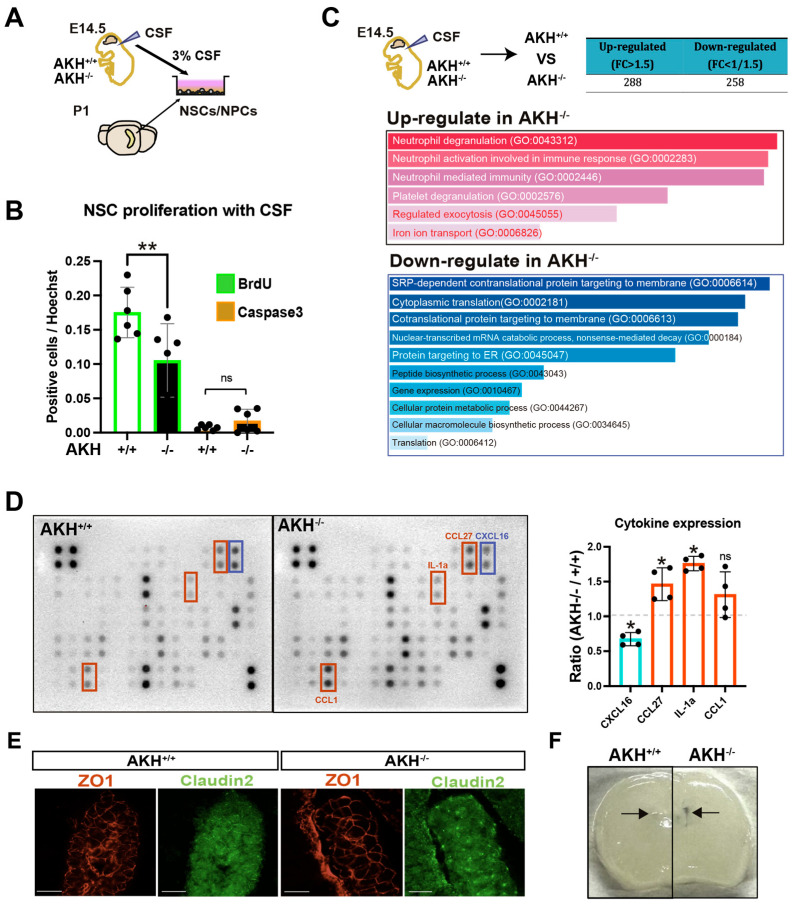
AKH deficiency impairs ChP barrier integrity and alters CSF composition. (**A**) Experimental scheme illustrating the addition of CSF collected from E14.5 embryos to NSCs/NPCs culture medium at a final concentration of 3%, followed by culture for 7 days. (**B**) BrdU was added 30 min before fixation, and BrdU^+^ proliferating cells and cleaved caspase-3^+^ apopyotic cells were quantified. AKH^+^/^+^: n = 6, AKH^−^/^−^: n = 6. *p* = 0.00915 (one-way ANOVA, two-sided, Tukey’s multiple comparison) (**C**) Non-targeted proteomics analysis of CSF pooled from seven AKH^+^/^+^ or AKH^−^/^−^ E14.5 embryonic mice. Differentially expressed proteins were defined using a fold-change threshold of 1.5, and enrichment analysis was performed using Enricher software. (**D**) Representative chemiluminescence-detected dot blot images. AKH^+^/^+^: n = 4, AKH^−^/^−^: n = 4. CXCL16: *p* = 0.0373, CCL27: *p* = 0.0145, IL-1α: *p* = 0.0445, CCL1: *p* = 0.0362. (**E**) Super-resolution microscopy images of the ChP from E14.5 embryos stained for tight junction markers ZO-1 and Claudin-2. Scale bars: 25 µm. (**F**) Evans blue permeability assay in P5 mice. Brains were collected 24 h after Evans blue administration, perfused with PBS, and sectioned at 300-µm thickness. Right arrow indicates the leaked Evans blue in the AKH^−^/^−^ LV. All data are presented as mean ± SD; * *p* < 0.05, ** *p* < 0.01. ns, not significant.

To determine whether AKH deficiency alters CSF-derived cues regulating NSCs growth, primary NSCs isolated from the hippocampus of P1 mice were cultured in medium supplemented with 3% CSF collected at E14.5 from either AKH^+^/^+^ or AKH^−^/^−^ embryos. After seven days, BrdU incorporation was assessed following a 30 min pulse. NSCs exposed to AKH^−^/^−^ CSF exhibited significantly reduced BrdU incorporation compared with those treated with AKH^+^/^+^ CSF (Figure 4A,B), indicating that CSF from AKH^−^/^−^ embryos contains antiproliferative factor(s).

To identify these factors, we performed label-free quantitative proteomic analysis of E14.5 CSF (Figure 4C). Among the 1184 detected proteins, 288 were upregulated (fold-change > 1.5) and 258 were downregulated (fold-change < 0.67) in AKH^−^/^−^ CSF. Gene ontology enrichment amalysis revealed a marked increase in neutrophil-related processes, such as neutrophil degranulation and activation, whereas down-regulated proteins were mainly associated with the translation, suggesting a shift toward a pro-inflammatory milieu.

Cytokine profiling using a RayBio^®^ C-Series Mouse Cytokine Array further confirmed elevated levels of inflammatory mediators, including CCL27 and interleukin-1 α (IL-1α), in CSF of AKH^−^/^−^ embryos (Figure 4D). Given that IL-1α is known to suppress NSCs proliferation, its increase provides a plausible mechanism for the proliferation defects observed both in vivo and in vitro [26]. Consistently, qPCR analysis revealed elevated expression of the IL-1 receptor (IL-1R) in GE-derived cells at E14.5 (Supplementary Figure S2I).

We next investigated whether the blood–CSF barrier is compromised in AKH^−^/^−^ mice. At E14.5, the tight-junction protein Claudin-2 was aberrantly upregulated and mis-localized in the ChP epithelium of AKH^−^/^−^ mice, whereas ZO-1 distribution remained unchanged (Figure 4E). Claudin-2 is a well-established regulator of paracellular permeability in brain barriers [27,28]. Consistantly, Evans-blue dye injected intraperitoneally at P5 exhibited increased leaked into the brain parenchyma of AKH^−^/^−^ mice compared with controls (Figure 4F).

Taken together, these findings suggest that AKH deficiency leads to Claudin-2 upregulation in the ChP, thereby compromising the integrity of the blood–CSF barrier (BCSFB) and allowing pro-inflammatory cytokines such as IL-1α to enter the CSF, where they may suppress NSCs proliferation.

### 3.5. Deficiency of AKH Leads to Behavioral Abnormalities

All behavioral assessments were conducted during the light phase to ensure experimental consistency and comparability between genotypes, in accordance with standard mouse behavioral testing paradigms.

To determine whether reduced NSCs proliferation and delayed cortical layer formation translate into behavioral deficits, male AKH^−^/^−^ and AKH^+^/^+^ littermates were examined at 10 weeks of age. A battery of established tests was applied to evaluate locomotor activity, anxiety-related behavior, motor coordination, and associative learning. AKH^−^/^−^ mice exhibit postnatal ventricular enlargement [13], a hallmark of hydrocephalus that is frequently associated with gait abnormalities and cognitive dysfunction.

**Figure 5:**
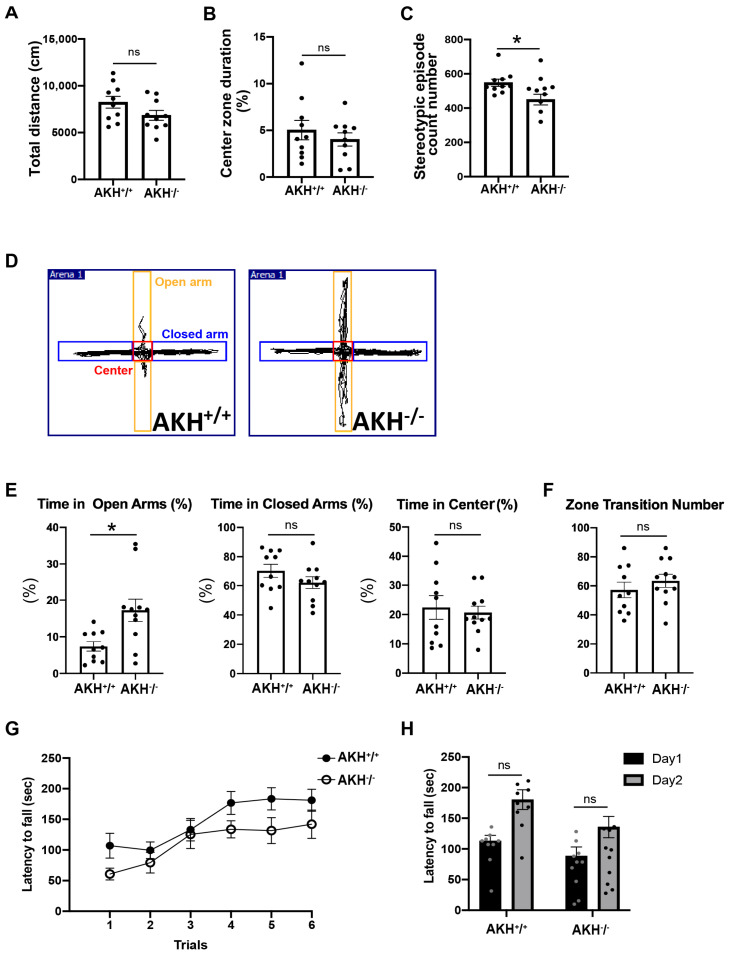
AKH^-/-^ mice exhibit anxiety-like behavior. (**A**–**C**) Locomotor activity and anxiety-like behavior assessed using the open field test. (**A**) Total distance traveled. *p* = 0.105. (**B**) Time spent in the center zone. *p* = 0.438. (**C**) Number of stereotypic episodes. *p* = 0.0181. (unpaired Student’s *t*-test). (**D**) Schematic representation of the elevated plus maze test. Open arms are shown in yellow, closed arms in blue, and center zone in red. (**E**,**F**) Anxiety-like behavior was evaluated using the elevated plus maze. (**E**) Latency to enter the open arms, time spent in the open arms, and number of open-arm entries. *p* values: *p* = 0.0100, *p* = 0.195, and *p* = 0.703, respectively. (**F**) Time spent in the closed arms (*p* = 0.378) (unpaired Student’s *t*-test). (**G**) Motor coordination and balance assessed using the Rota-rod test. Data were analyzed by two-way repeated-measures ANOVA. Genotype × time interaction, *p* = 0.688; time, *p* < 0.0001; genotype, *p* = 0.0544. (**H**) Latency to fall (sec) anayzed by two-way ANOVA, genotype × time interaction: *p* = 0.442, time: *p* = 0.0003, genotype: *p* = 0.0544. All data are presented as mean ± SD; * *p* < 0.05, ns, not siginificant. AKH^+^/^+^: n = 10, AKH^−^/^−^: n = 10.

To comprehensively assess locomotor activity, anxiety-related behavior, motor coordination, and associative learning, a battery of established behavioral tests was applied. Open-field testing revealed no genotype differences in total distance travelled or time spent in the center zone (Figure 5A,B). The number of stereotypic episodes in the open-field test was significantly reduced in AKH^−^/^−^ mice compared with controls, suggesting an alteration in repetitive behavioral patterns (Figure 5C). In contrast, performance in the elevated plus maze revealed a significantly increased latency to enter the open arms in AKH^−^/^−^ mice, suggesting enhanced anxiety-like behavior (Figure 5D–F).

Motor coordination and associative memory were largely preserved. Rotarod performance did not differ significantly between genotypes, although AKH^−^/^−^ mice exhibited a non-significant trend toward reduced latency to fall (Figure 5G,H). Similarly, contextual fear-conditioning induced comparable freezing levels in both groups (Supplementary Figure S3A–C), and light–dark box testing revealed no additional differences in anxiety-related behavior (Supplementary Figure S3A).

Taken together, these results indicate that AKH deficiency does not cause generalized impairments in locomotion or learning but instead leads to selective behavioral alterations characterized by heightened anxiety-like responses. These findings suggest preferential involvement of emotional regulation circuits rather than widespread motor or cognitive dysfunction.

Because behavioral abnormalities were observed in adult AKH^−^/^−^ mice, we next examined AKH expression in the adult brain. Immunohistochemical analyses revealed that AKH expression was undetectable in the ventricular region under basal conditions in 8-week-old mice (Supplementary Figure S4A). Given the essential role of AKH during neurogenesis, we next investigated whether AKH expression could be re-induced under conditions associated with adult neurogenic and reparative responses. To address this, we employed a transient middle cerebral artery occlusion (tMCAO) model, a well-established paradigm of injury-induced plasticity. The ipsilateral region was identified by robust CD68 immunoreactivity, confirming the presence of ischemic lesions (Supplementary Figure S4B). Notably, AKH expression was re-induced in the peri-infarct region following ischemic injury (Supplementary Figure S4C). Co-immunostaining revealed that overlap between AKH and the astrocytic marker S100β, indicating that reactive astrocytes represent a major cellular source of AKH in the adult ischemic brain (Supplementary Figure S4C).

### 3.6. AKH Contributes to the Prevention of Bacterial Spread in the Ventricles

**Figure 6:**
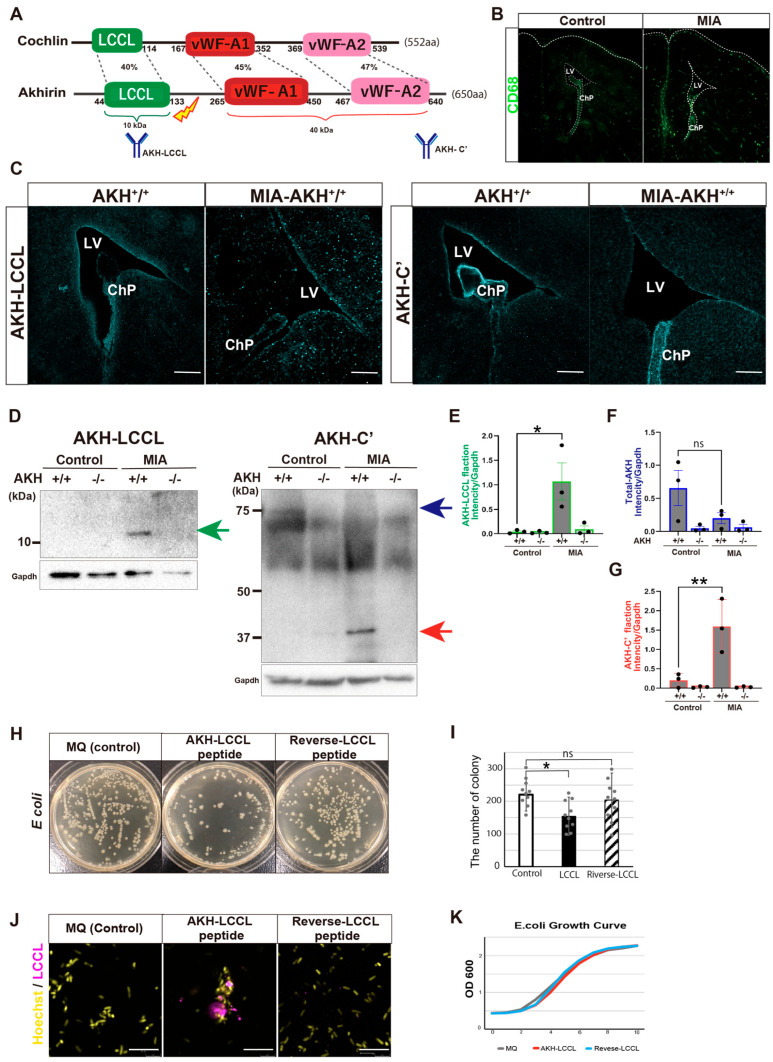
AKH contributes to preventing the spread of bacterial infection within the ventricles. (**A**) Schematic representation of the domain structures of AKH and Cochlin and the epitopes recognized by the generated antibodies. (**B**) IHC analysis using anti-CD68 in AKH^+^/^+^ and poly(i;c) injected AKH^+/+^ mice. (**C**) IHC analysis of AKH^+^/^+^ and MIA model mice using anti-AKH-LCCL and anti-AKH-C′ antibodies. (**D**) Western blotting analysis of CSF from AKH^+^/^+^ and MIA–AKH^+^/^+^ mice using anti-AKH-LCCL and anti-AKH-C′ antibodies. (**E**–**G**) Quantification of band intensities from (**D**). Blue indicates the total AKH, red indicates the C′-terminal fraction recognized by the anti-AKH-C′ antibody, and green indicates the LCCL-domain fraction recognized by the anti-AKH-LCCL antibody. (**H**,**I**) Representative *E. coli* DH5a culture plates and quantification of colony numbers. n = 10. (**J**), Immunocytechemistry of *E. coli* DH5a co-cultured with PBS, AKH-LCCL peptide, or reverse-LCCL peptide and stained with an anti-AKH-LCCL antibody. (**K**) OD600 measurement of *E. coli* DH5a cultured with AKH-LCCL peptide in LB medium. Data are presented as mean ± SD. n = 10. *p < 0.05, ** *p* < 0.01, Analyzed by two-way ANOVA, two-sided, Tukey’s multiple comparison). ns, not significant.

Cochlin is a molecule homologous to AKH (Figure 6A). Although Cochlin has primarily been studied in the context of hereditary hearing loss, recent studies have shown that its LCCL domain is proteolytically released during bacterial infection, where it directly binds bacteria and stimulates innate immune responses by recruiting macrophages, thereby preventing infection-induced tissue damage in the inner ear [29]. While Cochlin is not expressed in the brain, the structural similarity between Cochlin and AKH, together with the ventricular localization of AKH adjacent to NSCs, led us to hypothesize that AKH fulfills a comparable immune-protective function in the developing brain.

To test this hypothesis, we employed a maternal immune activation (MIA) model of prenatal inflammation induced by poly(i;c) injection, in which systemic immune challenge during gestation elicits innate immune response in the embryo. Embryos were collected at E14.5 and examined for inflammation-induced changes in AKH processing and localization (Figure 6A).

Immune activation in the embryonic brain was first confirmed by a significant increased in CD68^+^ immune cells (Figure 6B). To determine whether AKH undergoes proteolytic cleavage in response to maternal immune challenge, we generated an antibody specific to the LCCL domain of AKH, in addition to an antibody recognizing the C-terminal domain (Figure 6A). Immunostaining revealed that LCCL immunoreactivity became more diffuse throughout the ventricular region in MIA embryos, whereas the C-terminal AKH signal was markedly reduced at the ventricular surface and largely confined to the ChP epithelium (Figure 6C).

Western blot analysis of embryonic CSF further confirmed the presence of cleaved AKH fragments, including bands corresponding to the LCCL and vWF domains (Figure 6D–G), indicating that maternal immune challenge promotes AKH cleavage and the release of its functional domains into the CSF.

Based on the antibacterial activity reported for the Cochlin-LCCL domain, we next investigated whether the AKH-LCCL peptide exhibits similar properties. A synthetic AKH-LCCL peptide was incubated with *Escherichia coli* DH5a (*E. coli* DH5a), resulting in significant reduction in bacterial colony formation (Figure 6H,I). Immunodetection assays demonstrated direct binding of the AKH-LCCL peptide to *E. coli* DH5a, whereas a reverse-sequence control peptide showed no detectable binding (Figure 6J). In contrast, bacterial growth curve assessed by optical density measurements were not significantly altered (Figure 6K), suggesting that AKH-LCCL suppresses colony formation by interfering with bacterial adhesion rather than bacterial proliferation.

Together, these results demonstrate that AKH undergoes proteolytic activation in response to prenatal immune stress and functions as an innate immune effector in the embryonic brain. By binding invading bacteria and limiting their dissemination within the ventricles, AKH may protect the NSC niche during critical stages of brain development. 

## 4. Discussion

Our study identifies AKH as a multifunctional protein localized to the ventricular zone that links early neuroimmune homeostasis with long-term brain function. We demonstrate that the loss of AKH during mid-gestation triggers a cascade of events, including reduced NSCs/NPCs proliferation, delayed neuronal and glial differentiation, and a shift toward a pro-inflammatory CSF milieu. These developmental alterations ultimately result in anxiety-like and stereotypic behaviors in adulthood that resemble ASD traits [30,31]. These findings align with the developmental origins of health and disease (DOHaD) concept, which posits that transient intrauterine inflammatory perturbations during critical periods of intrauterine development can exert long-lasting effects on brain function and behavior [32,33,34].

Mechanistically, MIA experiments revealed that AKH undergoes proteolytic cleavage under inflammatory stress, releasing an LCCL-containing fragment that binds directly to bacteria and suppresses colony formation. This fragment thus functions as an innate immune “gatekeeper” within the embryonic ventricles. We propose that insufficient AKH activity compromises this intraventricular defense, permitting the accumulation of inflammatory mediators such as IL-1α in the CSF, disrupt the neurogenic niche, and predispose the developing brain to neurobehavioral dysfunction later in life [35].

Because AKH is abundantly expressed along the apical surface of ChP epithelial cells, we predicted that AKH is secreted into the CSF upon synthesis, with a fraction remaining associated with the apical surface of ventricular NSCs/NPCs layers. This model is supported by two observations: (i) AKH immunostaining appears patchy rather than uniform along the apical NSCs/NPCs surface [13], and (ii) AKH is largely absent from the lower LV region where CSF turnover is limited. Upon MIA, proteolytic cleavage of AKH liberating the LCCL domain, which binds to bacteria and limits their intraventricular dissemination. Collectively, these findings suggest that AKH functions as an intraventricular immune barrier that protects the developing NSC/NPC niche from microbial invasion and preserves niche homeostasis during a critical window of brain development.

The AKH-LCCL domain may also bind to and inhibit a broader spectrum of pathogens beyond *E. coli* DH5a. Notably, *Pseudomonas aeruginosa* and influenza virus have been reported to interact directly with the LCCL domain of Cochlin, a protein homologous to AKH [29]. Several bacteria and viruses enhance their infectivity by secreting glycosaminoglycan (GAG)-degrading enzymes that modify host GAGs to facilitate cellular entry [36]. GAGs, including heparan sulfate, heparin, and chondroitin sulfate, are sulfated polysaccharides abundantly present on cell surfaces and within the ECM [37]. These molecules function not only as attachment platforms for pathogen but also as recognition substrates for innate immune cells [38]. In the context of influenza infection, both heparan sulfate and chondroitin sulfate have been implicated in host defense mechanisms [39,40]. Importantly, the Cochlin-LCCL domain has been shown to bind these sulfated GAGs, thereby inhibiting bacterial adherence and modulating innate immune activation [41,42]. Because Cochlin is not expressed in the brain, it is plausible that the LCCL domain of AKH, expressed within the embryonic ventricular system, serves a functionally analogous immune-protective role during brain development.

The altered CSF composition observed in AKH^−^/^−^ mice, particularly the elevation of IL-1α, is consistent with previous reports showing that pro-inflammatory cytokines suppress NSCs proliferation and disrupt the balance between neuronal and glial differentiation. We demonstrate that AKH deficiency during embryonic development disrupts cortical layer formation and result in anxiety-like behavior in adulthood. These findings align with the DOHaD framework, which posits that molecular and environmental perturbations during critical developmental windows can program long-term brain function and behavioral outcomes [43,44]. Our data suggests that AKH contributes to an intraventricular immune barrier that protects the neurogenic niche from inflammatory stress and microbial challenges. Loss of this protective function may permit excessive innate immune activation, leading to impaired neurogenesis and gliogenesis and to persistent alterations in neural circuits that regulate emotional regulation. Elucidating AKH’s role within this developmental context provides mechanistic insight into how early immune imbalance confers long-term behavioral vulnerability and highlights AKH-mediated pathways as potential targets for early intervention in stress-related and neuropsychiatric disorders.

We previously demonstrated that *AKH* mRNA is specifically expressed in the SVZ and hippocampus regions that harbor NSC/NPC niches from E17.0 [15]. Notably, *AKH* expression declines and becomes undetectable by P20, coinciding with the physiological tapering of postnatal neurogenesis in mice [15,16]. Although the precise relationship between AKH expression and NSCs proliferation remains to be fully elucidated, this temporal overlap suggests that AKH function as a transient immune regulator, protecting proliferating NSCs from infection during a developmentally vulnerable period critical for brain formation. Interestingly, AKH expression is reactivated in the adult spinal cord following injury, where it localizes to NSC/NPC niches [16]. Extending this observation to the adult brain, we employed a tMCAO model to induce focal brain injury. AKH was re-expressed in the ipsilateral hemisphere (Supplementary Figure S4C), indicating that AKH expression can be re-induced in the adult brain in response to injury. Notably, AKH immunoreactivity overlapped with the astrocyte marker S100β, suggesting that reactive astrocytes at the lesion site are a source of AKH expression and possibly secretion. Unexpectedly, AKH expression in the injured adult brain was not associated with NSCs/NPCs populations but was instead localized to reactive astrocytes. This finding suggests that AKH may assume a distinct role in the adult brain, particularly in the context of inflammation and tissue injury. Importantly, whether astrocyte-derived AKH exerts protective, regenerative, or inflammation-limiting effects remains to be determined. Future studies using tMCAO models in AKH^−^/^−^ mice will be essential to elucidate additional and potentially unexpected roles of AKH in the injured adult brain, including its contribution to secondary injury, glial responses, and endogenous repair mechanisms.

Given the antimicrobial activity of the AKH-LCCL domain and its ventricular localization during development, we hypothesize that AKH re-expression at injury sites functions as a local immune barrier, limiting bacterial invasion and contributing to tissue protection. In the context of adult CNS regeneration, it is important to note that many of the spatial and molecular cues that guide neural development during ontogenesis are absent or substantially altered in the mature brain [45,46,47]. Nevertheless, accumulating evidence indicates that elements of developmental programs can be partially reactivated following injury, particularly within specialized niches such as the SVZ or peri-lesional areas [48,49]. Our observation that AKH expression is re-induced in the adult brain following ischemic injury raises the possibility that AKH may contribute to shaping the regenerative microenvironment, rather than directly recapitulating its developmental functions. Given its extracellular localization and immunomodulatory properties, AKH may influence adult repair processes by limiting inflammation, stabilizing the local niche, or modulating interactions between neural and glial cells. However, whether AKH actively promotes regeneration or instead serves primarily as a protective factor that constrains secondary damage remains an open question and warrants further investigation. Together, these findings support a model in which AKH exerts context-dependent functions, acting as a niche-protective factor during development and as an injury-induced innate immune modulator in the adult brain. This dual role highlights the broader relevance of AKH in maintaining CNS integrity throughout life.

AKH appears to function as an immunological barrier in the embryonic brain, a developmental stage at which the immune system is immature and the BCSFB is not yet fully established. Its re-expression at sites of injury in adulthood suggests that AKH may play a conserved role in protecting the neurogenic niche under both developmental and pathological conditions. While our previous work positioned AKH primarily as a local niche-associated factor regulating NSCs proliferation [15,16], the current study expands this view by identifying AKH as a previous unrecognized innate immune modulator that limits inflammation-induced disruption of neurogenesis. Given its highly restricted temporal expression pattern and context-dependent dual functionality, AKH may represent a promising molecular target for safeguarding brain development and promoting tissue responses following CNS injury.

One limitation of the present study is the use of a whole-body AKH knockout model, which may involve systemic compensatory mechanisms or indirect effects that cannot be fully excluded. Moreover, although AKH appears to be a highly adhesive extracellular protein, its specific binding partners or receptors have not yet been identified. Despite our attempts, their identification proved technically challenging. Future studies employing conditional and cell type-specific AKH deletion, particularly in the ChP or astrocytes, will be essential to delineate the precise cellular sources and context-dependent roles of AKH in the CNS. Despite these limitations, our findings underscore the physiological importance of AKH in maintaining immune balance at the brain–CSF interface and open new avenues for developing AKH-based therapeutic strategies for neurodevelopmental and neuroinflammatory disorders.

## 5. Conclusions

AKH is a secreted factor that contributes to the formation and maintenance of the NSC/NPC niche during brain development. In this study, we demonstrated that AKH protein localizes to the apical surface of ChP epithelial cells and the ventricular wall in the embryonic mouse brain at E14.5, where it supports NSC/NPC niche homeostasis via the CSF. Loss of AKH resulted in elevated levels of inflammatory cytokines in the CSF and disrupted the balance between NSCs/NPCs proliferation and differentiation. Furthermore, we show that the LCCL domain of AKH directly binds to bacteria and restricts their dissemination. Together, these findings suggest that AKH functions as a molecular barrier within the developing brain, compensating for the immature innate immune defenses and maintaining the physiological microenvironment required for proper neurogenesis.

## Data Availability

The original contributions presented in this study are included in the article/Supplementary Material. Further inquiries can be directed to the corresponding author(s).

## Supplementary Materials

The following supporting information can be downloaded at: https://www.mdpi.com/article/10.3390/cells15020151/s1.

## Abbreviations

AKH	Akhirin
NSCs	Neural stem cells
CSF	Cerebrospinal fluid
LCCL	Limulus factor C, cochlear protein, and late gestation lung protein domain
NPCs	Neural progenitor cells
SVZ	Subventricular zone
ECM	Extracellular matrix
ChP	Choroid plexus
BCSFB	Blood-CSF barrier
PBS	phosphate-buffered saline
PFA	Paraformaldehyde
BrdU	5-bromo-2′-deoxyuridine
Caspase3	Cysteine-aspartic protease 3
CD68	Cluster of differentiation 68
CD206 (MMR)	Macrophage mannose receptor
Claudin-2	Claudin family member 2
Flk1	Fetal liver kinase 1
GFAP	Glial fibrillary acidic protein
Iba1	Ionized calcium-binding adaptor molecule 1
IB4	Isolectin B4
P2RY12	Purinergic receptor P2Y12
PHH3	Phospho-histone H3
S100β	S100 calcium-binding protein β
Sox2	SRY-box transcription factor 2
Tbr1	T-box brain transcription factor 1
ZO1	Zonula occludens-1
LV	Lateral ventricle
EGF	Epidermal growth factor
FGF	Fibroblast growth factor
DMEM	Dulbecco’s modified eagle medium
IHC	Immunohistochemistry
CMF	calcium-magnesium free phosphate buffer
KLH	Keyhole limpet hemocyanin
HRP	Horseradish peroxidase
GE	Ganglionic eminence
NaCl	Sodium Chloride
SDS	Sodium dodecyl sulfate
Tris-HCl	Tris(hydroxymethyl)aminomethane hydrochloride
RIPA	Radioimmunoprecipitation assay (buffer)
NP-40	Nonidet P-40
PVDF	Polyvinylidene difluoride
TBS-T	Tris-buffered saline containing Tween-20
GAPDH	Glyceraldehyde-3-phosphate dehydrogenase
DOHaD	Developmental Origins of Health and Disease
MIA	Maternal immune activation
GAG	Glycosaminoglycan
tMCAO	transient middle cerebral artery occlusion
