# Supplementary material for: Akhirin Functions as an Innate Immune Barrier to Preserve Neurogenic Niche Homeostasis During Mouse Brain Development
Source: Cells. 2026 Jan 14;15(2):151. doi: 10.3390/cells15020151 (PMC12839204; doi:10.3390/cells15020151)
Supplement: Supplementary file 1 [file cells-15-00151-s001.zip › cells-4010137-supplementary.pdf]

Abbreviations

The following abbreviations are used in this manuscript:

AKH	Akhirin
NSCs	Neural stem cells
CSF	Cerebrospinal fluid
LCCL	Limulus factor C, cochlear protein, and late gestation lung protein domain
NPCs	Neural progenitor cells
SVZ	Subventricular zone
ECM	Extracellular matrix
ChP	Choroid plexus
BCSFB	Blood-CSF barrier
PBS	phosphate-buffered saline
PFA	Paraformaldehyde
BrdU	5-bromo-2′-deoxyuridine
Caspase3	Cysteine-aspartic protease 3
CD68	Cluster of differentiation 68
CD206 (MMR)	Mannose receptor, macrophage
Claudin-2	Claudin family member 2
Flk1	Fetal liver kinase 1
GFAP	Glial fibrillary acidic protein
Iba1	Ionized calcium-binding adaptor molecule 1
IB4	Isolectin B4
P2RY12	Purinergic receptor P2Y12
PHH3	Phospho-histone H3
S100β	S100 calcium-binding protein β
SOX2	SRY-box transcription factor 2
Tbr1	T-box brain transcription factor 1
ZO1	Zonula occludens-1
LV	Lateral ventricle
EGF	Epidermal growth factor
FGF	Fibroblast growth factor
DMEM	dulbecco's modified eagle medium
IHC	Immunohistochemistry
CMF	calcium-magnesium free phosphate buffer
KLH	Keyhole limpet hemocyanin
HRP	Horseradish peroxidase
GE	Ganglionic eminence
NaCl	Sodium Chloride
SDS	Sodium dodecyl sulfate
Tris-HCl	Tris(hydroxymethyl)aminomethane hydrochloride
RIPA	Radioimmunoprecipitation assay (buffer)
NP-40	Nonidet P-40
PVDF	Polyvinylidene difluoride
TBS-T	Tris-buffered saline containing Tween-20
GAPDH	Glyceraldehyde-3-phosphate dehydrogenase
DOHaD	Developmental Origins of Health and Disease
MIA	Maternal immune activation
GAG	Glycosaminoglycan
tMCAO	transient middle cerebral artery occlusion

Supplementary figure S1

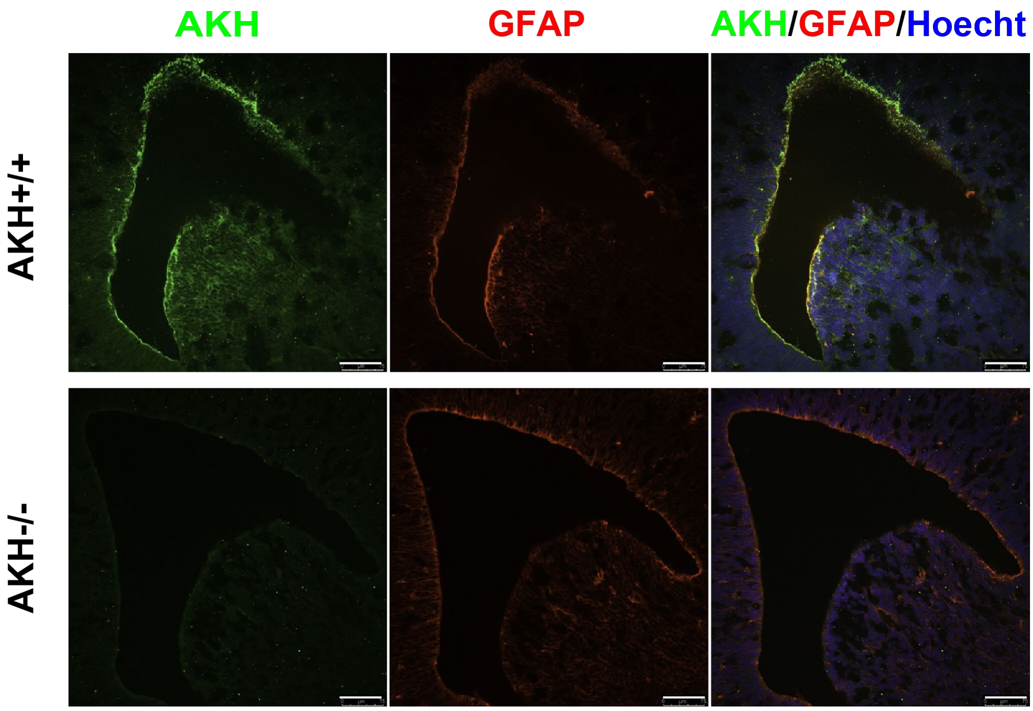

Supplementary figure S1

IHC image of AKH, GFAP, Hoechst of $AKH^{+/+}$ and $AKH^{-/-}$. Scale bars: 75 μm.

Supplementary figure S2

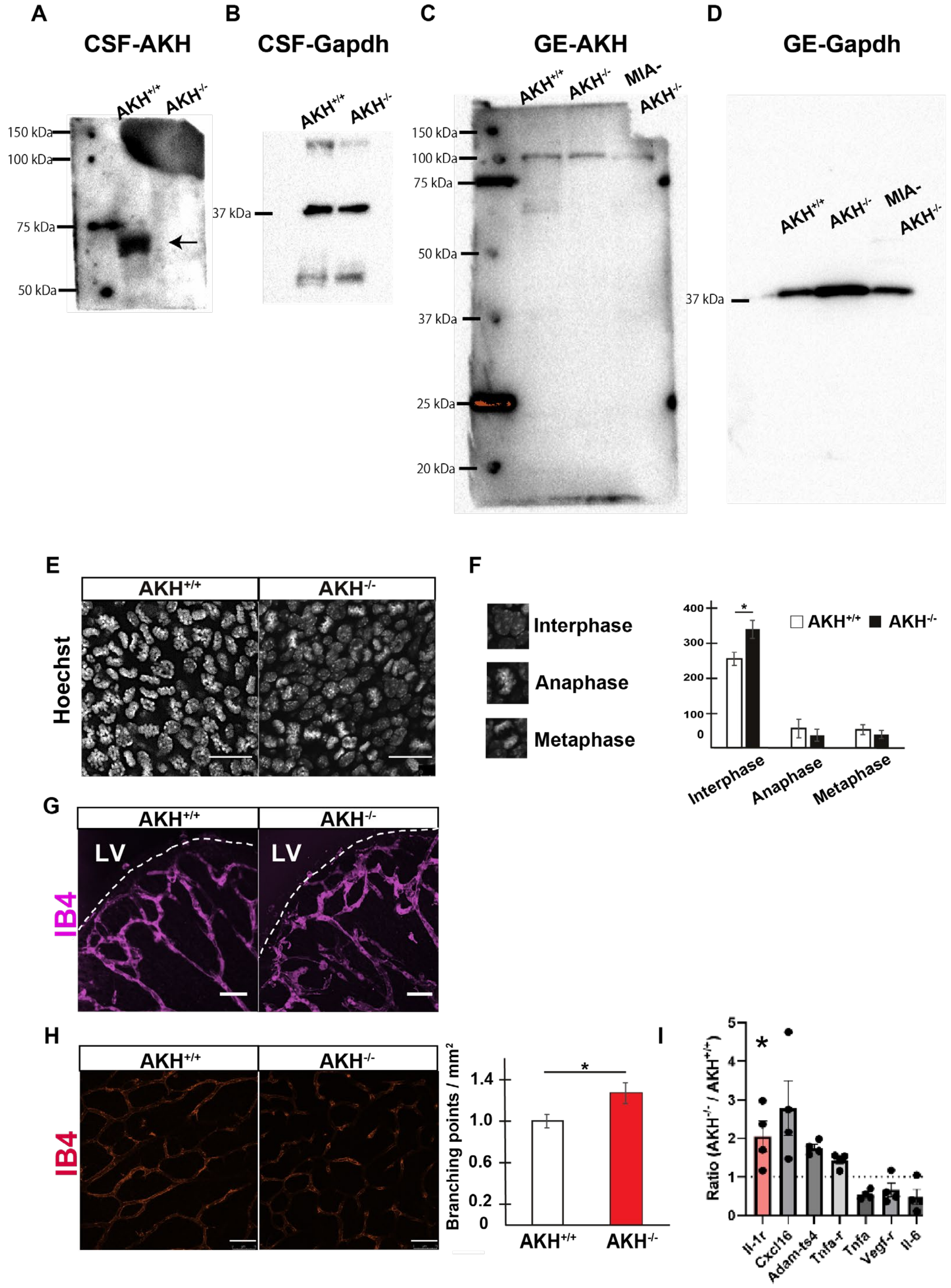

Supplementary figure S2

(A-D) Western blotting was performed to verify the molecular weight of AKH in CSF and GE. Arrow in (A) indicates the AKH protein band. (E) Hoechst signals at VZ of AKH$^{+/+}$ and AKH$^{-/-}$ E14.5 mouse. (F) Quantification of each cell cycle phase cells. (G) IHC image of IB4-positive blood vessel at GE of AKH$^{+/+}$ and AKH$^{-/-}$. (H) IHC image of IB4-positive blood vessel at VZ of AKH$^{+/+}$ and AKH$^{-/-}$. (I) Quantifications of cytokines of AKH$^{+/+}$ and AKH$^{-/-}$. *$p < 0.05$, (Student's t-test). All data are presented as mean ± SD. *$p < 0.05$, (Student's t-test).

Supplementary figure S3

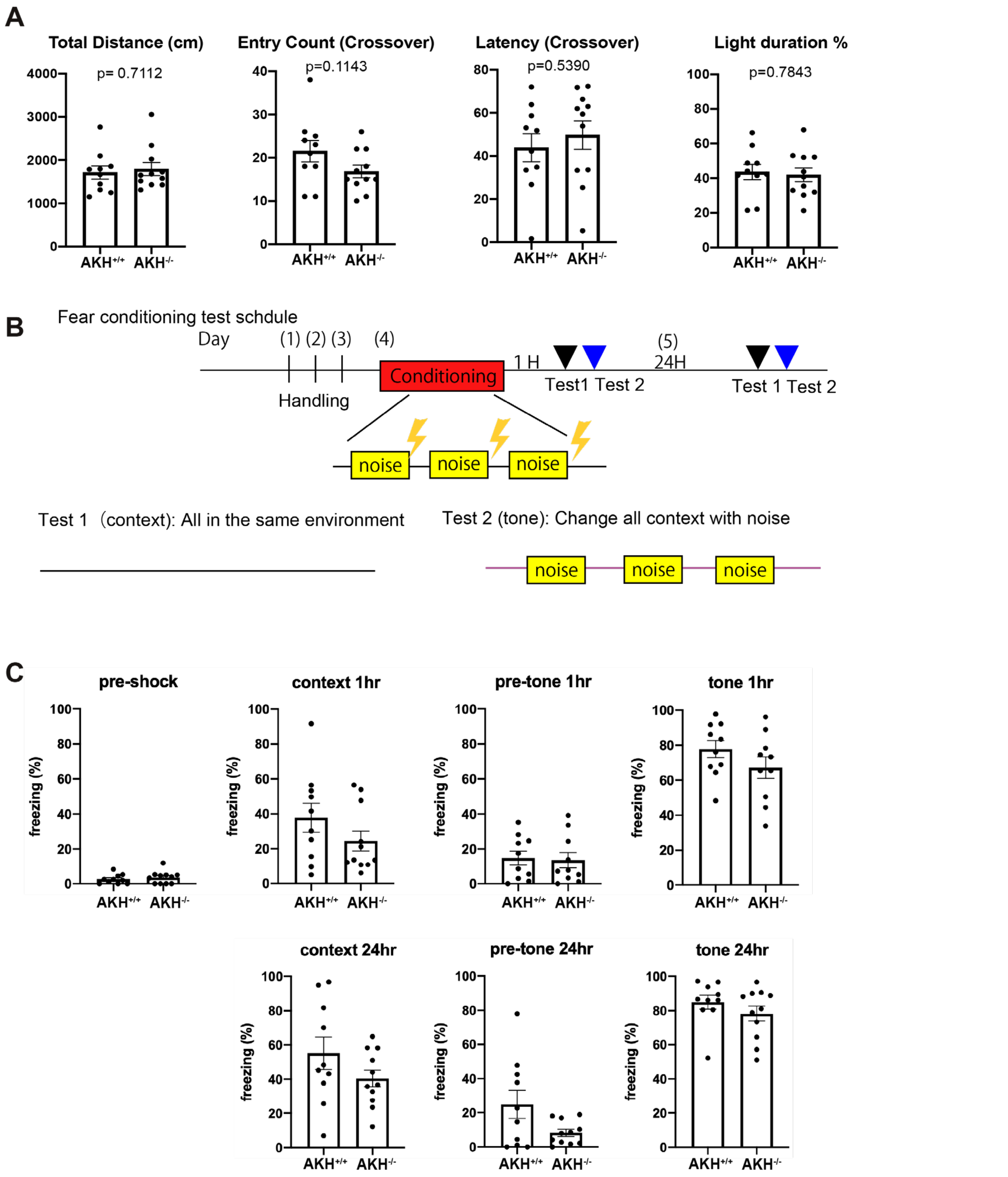

Supplementary figure S3

(A) The graphs show the total distance, entry count to light field, latency and duration of light field of light and dark test. (B) Fear conditioning test schedule. (C) The quantification of time of freezing (%) of each test. No test revealed a statically significant difference (unpaired Student's t-test).

Supplementary figure S4

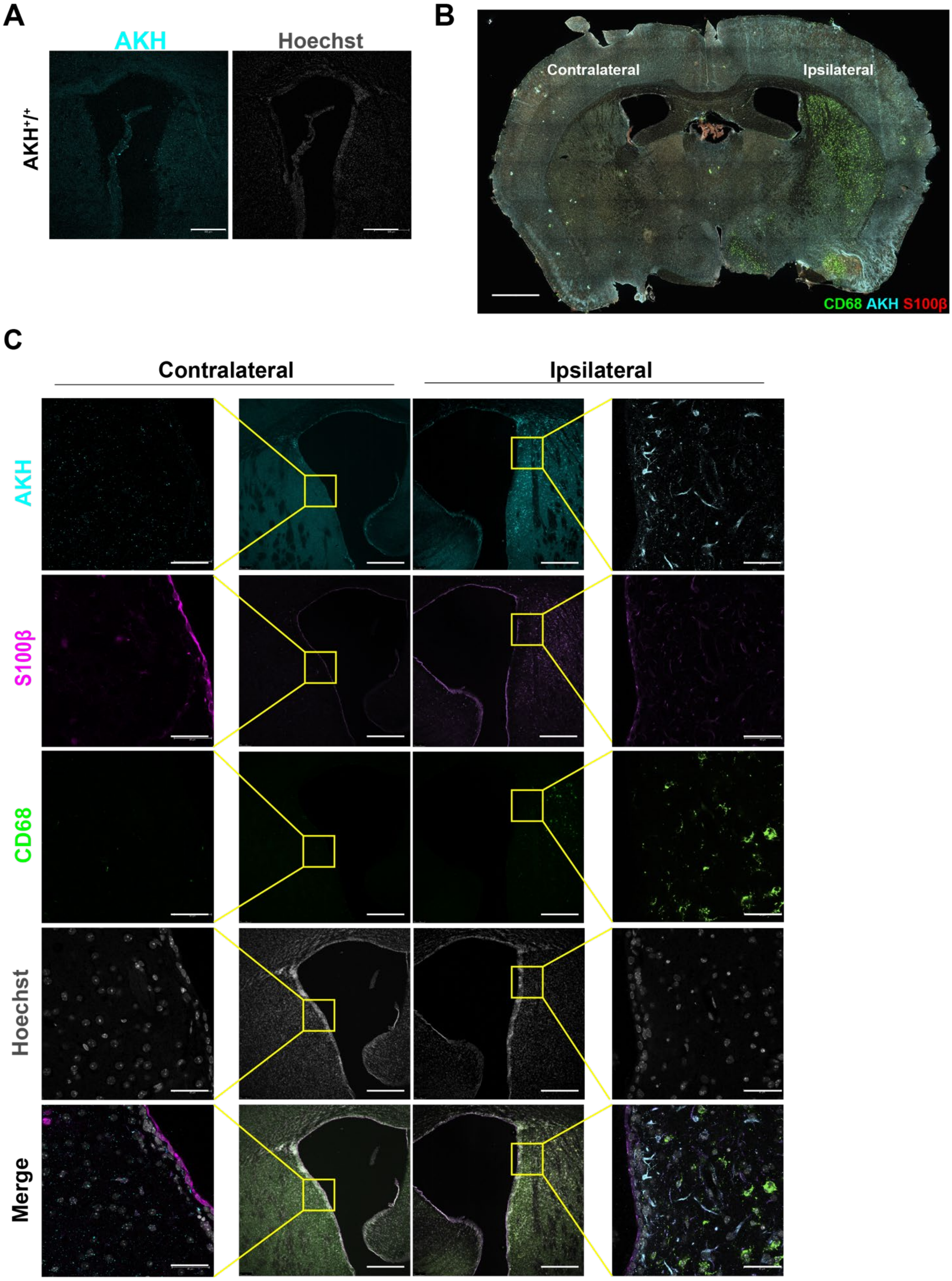

Supplementary figure S4

(A) The images show the expression of AKH and Hoechst of 8 weeks AKH^{+}/$^{+}$. Scale bars: 300 μm. (B) The image shows contralateral side and ipsilateral side in tMCAO treated brain section. Scale bars: 1mm. (C) The images show AKH, S100β, CD68 and Hoechst expression each. Scale bars: 100 μm (center), 40 μm (High-magnification image).
